# Trade-offs between sociality and gastrointestinal parasite infection in the context of a natural disaster

**DOI:** 10.1016/j.anbehav.2024.03.002

**Published:** 2024-04-01

**Authors:** Melissa A. Pavez-Fox, Carla M. Escabi-Ruiz, Jordan D. A. Hart, Josue E. Negron-Del Valle, Daniel Phillips, Angelina Ruiz-Lambides, Samuel E. Bauman, Cayo Biobank Research Unit, Melween I. Martinez, Michael J. Montague, Michael L. Platt, James P. Higham, Noah Snyder-Mackler, Lauren J. N. Brent

**Affiliations:** a Centre for Research in Animal Behaviour, University of Exeter, Exeter, U.K.; b School of Psychology and Neuroscience, University of St Andrews, St Andrews, U.K.; c Department of Neuroscience, University of Pennsylvania, Philadelphia, PA, U.S.A.; d Center for Evolution and Medicine, Arizona State University, Temple, AZ, U.S.A.; e Caribbean Primate Research Center, University of Puerto Rico, San Juan, Puerto Rico; f Department of Human Behavior, Ecology and Culture, Max Planck Institute for Evolutionary Anthropology, Leipzig, Germany; g Departments of Anthropology, University of Pennsylvania, Philadelphia, PA, U.S.A.; h Departments of Psychology, University of Pennsylvania, Philadelphia, PA, U.S.A.; i Departments of Marketing, University of Pennsylvania, Philadelphia, PA, U.S.A.; j Department of Anthropology, New York University, New York, NY, U.S.A.; k School of Life Sciences, Arizona State University, Temple, AZ, U.S.A.; l School for Human Evolution and Social Change, Arizona State University, Temple, AZ, U.S.A.

**Keywords:** hurricane, infection, sociality, social network, rhesus macaque

## Abstract

Parasites and infectious diseases constitute important challenges particularly for group-living animals. Social contact and shared space can both increase parasite transmission risk, while individual differences in social capital can help prevent infections. For example, high social status individuals and those with more or stronger affiliative partnerships may have better immunity and, thus, lower parasitic burden. To test for health trade-offs in the costs and benefits of sociality, we quantified how parasitic load varied with an individual’s social status, as well as with their affiliative relationships with weakly and strongly bonded partners, in a free-ranging population of rhesus macaques, *Macaca mulatta*. We found that high status was associated with a lower risk of protozoa infection at older ages compared to younger and low-status animals. Social resources can also be protective against infection under environmentally challenging situations, such as natural disasters. Using cross-sectional data, we additionally examined the impact of a major hurricane on the sociality - parasite relationship in this system and found that the hurricane influenced the prevalence of specific parasites independent of sociality. Overall, our study adds to the growing evidence for social status as a strong predictor of infection risk and highlights how extreme environmental events could shape vulnerability and resistance to infection.

Parasites and infectious diseases are one of the major costs associated with group living. High population density and social interactions can increase parasite infection, particularly of parasites that rely on physical contact between hosts (Altizer et al., 2003). However, not all individuals in a group are equally likely to be infected. One potential driver of variation in exposure and susceptibility to infection is differentiated patterns of interactions (i.e. not all group members interact with all others; Duboscq et al., 2019). For instance, it is now well established that social partners are not only a potential source of infection but they can also impact positively their partner’s health and fitness (Silk, 2007; Snyder-Mackler et al., 2020). Recognizing how group-living animals manage the significant trade-off between the costs and benefits of social interactions can bring us closer to understanding the evolution of social relationships. Factors to consider in this trade-off include the types of interactions engaged in, the quality of relationships that emerge from those interactions, and the extent to which social hierarchies dictate inequalities in individual access to resources.

Affiliative social behaviours can have opposing effects on infection risk that may depend on the type of interaction. Grooming, a common affiliative behaviour observed in mammals and birds (preening), involves direct social contact and can increase the likelihood of infection by directly transmitted parasites. For example, individuals with more grooming partners or that engage more often in grooming interactions are more likely to be infected with nematodes in Japanese macaques, *Macaca fuscata* (MacIntosh et al., 2012), spider monkeys, *Ateles hybridus* (Rimbach et al., 2015) and savannah baboons, *Papio cynocephalus* (Habig et al., 2019). However, grooming has also been linked to health benefits by directly removing ectoparasites (Akinyi et al., 2013; Duboscq et al., 2016). Grooming is also considered a key behaviour by which animals establish social relationships (Carter & Leffer, 2015; Forand & Marchinton, 1989; Henzi & Barrett, 1999; Kutsukake & Clutton-Brock, 2006), which can themselves provide indirect health benefits by increasing access to food or shelter (Samuni et al., 2018), or by preventing injuries (Pavez-Fox et al., 2022), which can, in turn, promote immunity and resistance to parasites (Balasubramaniam et al., 2016; Pavez-Fox et al., 2021). Similar to grooming, close spatial proximity of individuals might facilitate the transmission of parasites, especially those that can survive for extended periods in the environment. For instance, belonging to the same social group and sharing space predicted bacterial and protozoa infection in Verreaux’s sifakas, *Propithecus verreauxi* (Springer et al., 2016) and Grant’s gazelles, *Nanger granti* (Williams et al., 2017), respectively. But spatial proximity is also commonly considered to reflect social tolerance among groupmates; thus, it could alternatively reduce the susceptibility and exposure to infection. For example, social tolerance might provide animals direct access to the resources necessary to maintain optimal health, access to resources of better quality (i.e. uncontaminated food; Carne et al., 2011; Dale et al., 2017; Fichtel et al., 2018; Springer et al., 2016) or information that facilitates access to such resources (Evans et al., 2020). All these examples highlight the trade-offs between the benefits and the infection costs of different types of interactions with conspecifics.

Yet, the type of interaction is not the only factor influencing an individual’s exposure to and ability to cope with communicable diseases; the quality of their relationships with others may play a key additional role. A growing body of research has highlighted the putative importance of the strength of social relationships on an individual’s fitness (Ellis et al., 2019; McFarland et al., 2017; Schulke et al., 2022) with potential implications for parasite transmission. Having many social partners with whom infrequent interactions occur (i.e. weak relationships) may pose a higher risk of transmission by increasing the diversity of hosts with whom an animal interacts (Vanderwaal et al., 2016). Strong relationships, where stable social partners frequently interact, may also increase the risk of parasite transmission because of longer exposure time (Muller-Klein et al., 2019). Depending on the context, individuals may prioritize specific relationship types as a way to compensate for the costs associated with the social transmission of parasites. For example, weak affiliative relationships may be beneficial in adverse environmental conditions and/or when resources are limited. In macaque species having more social partners has been associated with enhanced social thermoregulation (Campbell et al., 2018) and, presumably, heat stress avoidance (Testard et al., 2021) when facing harsh winters or a major hurricane, respectively. Strong affiliative relationships can also promote health when expensive returns are required. In vampire bats, *Desmodus rotundus*, for instance, individuals are more likely to donate a blood meal to bats with whom they groom more frequently (Carter & Leffer, 2015). Therefore, both strong and weak affiliative relationships could potentially compensate for the health costs of infectious diseases derived from sociality, but their relative importance may differ depending on the environmental conditions.

Another key component of social capital that might also have competing effects on infection risk are dominance hierarchies. On the one hand, social status may determine inequalities in access to resources, where individuals higher in the hierarchy often have an advantage compared to low-status individuals (Clutton-Brock & Huchard, 2013). Priority of access to resources for high-status animals might translate into better health and, therefore, reduced susceptibility to infection if associated with higher food intake, allowing the allocation of more resources to immunity (Sapolsky, 2005). On the other hand, high social status has been associated with a higher risk of infection (Habig & Archie, 2015). This has been suggested to be driven by dominant individuals being preferred affiliative partners (MacIntosh et al., 2012), their priority of access to mates, which may lead to higher parasite exposure due to more frequent social contact (Habig et al., 2018), and higher susceptibility due to the high energetic demand of reproduction (Smyth & Drea, 2016; Smyth et al., 2016), which often affects more males than females due to testosterone-mediated immunosuppression (Klein, 2000). Thus, how dominance hierarchies impact infection risk is likely to vary depending on the study system, an individual’s sex and how the inequalities it entails translate into immunity differences.

Growing evidence has contributed to our understanding of the associations between affiliative relationships, social status and infectious diseases (Briard & Ezenwa, 2021; Duboscq et al., 2019; Sadoughi et al., 2022). Yet results are mixed, and we are still far from a thorough comprehension of the contexts under which social interactions constitute a risk, or act as a buffer against parasite infections. For instance, the ecological context can shape not only the distribution and abundance of parasite species (Altizer et al., 2006) but also the aggregation patterns of individuals (Testard et al., 2021), both with potential consequences for infection risk (Sweeny et al., 2021). Further efforts to disentangle some of the factors that may influence the link between sociality and infection, including the types of social interactions involved in parasite transmission, the role of different affiliative relationships in buffering infection risk along with individual differences in exposure and susceptibility with social status, are therefore required. Moreover, the association between sociality and infectious diseases is likely to be modulated by other individual attributes such as sex and age, which can further contribute to differences in susceptibility due to immunosuppression (Klein, 2000) and immunosenescence (Ginaldi et al., 2001), or to differences in exposure due to mating effort (Habig & Archie, 2015) and social selectivity with age (Albery et al., 2023; Siracusa et al., 2022). All these trade-offs must be particularly relevant in the context of extreme environmental events, which may cause dramatic changes in the environment and in the dynamics of parasite transmission (Albery et al., 2021).

Here, we studied whether social and ecological variation predicted gastrointestinal parasite infection in a free-ranging population of rhesus macaques. Monkeys in this population self-organize into groups and interact spontaneously with each other, allowing us to explore the consequences of natural variation in social behaviour for infection risk. A natural disaster, Hurricane Maria, hit this population in 2017 causing dramatic changes in the environment including a massive decline in vegetation (63%) and important changes in aggregation patterns of individuals (Testard et al., 2021), which provides a unique opportunity to explore how changes in ecological conditions shape social and parasite dynamics. Using opportunistically collected cross-sectional data we first explored whether an individual’s social status predicted infection risk independently or dependent on the effect of sex, age and the hurricane. Given that social status might determine access to better or uncontaminated resources and enhanced immunity to fight parasites (Sanchez Rosado et al., 2024; Sapolsky, 2005) we expected that high-status individuals would be less likely to be parasitized, especially under the context of the hurricane. We also expected that sex and age might mediate this relationship, with older animals and/or males at higher risk due to immunosenescence (Ginaldi et al., 2001) and testosterone-mediated immunosuppression (Tidiere et al., 2020). Next, we explored trade-offs between infection risk and health benefits associated with affiliative relationships. To try to disentangle the roles of different types of affiliative social interaction (i.e. grooming and proximity) and the strength of relationships (i.e. weak and strong) in infection risk, we tested whether the number of weak grooming partners, weak proximity partners or the frequency of interaction with strong grooming partners predicted infection risk in this population. Given the complexity of the trade-offs detailed above, we did not have clear predictions for these analyses. However, given that social partners (Pavez-Fox et al., 2021) have been linked with immune benefits in this system, we expected in a general sense that individuals with more social capital, in the form of either weak partners or strong relationships, would be less susceptible to infection, while the type of interaction involved might determine the types of parasites found.

## METHODS

### Ethical Note

All research followed the ASAB/ABS Guidelines for the ethical treatment of animals. All data were collected following protocols approved by the Institutional Animal Care and Use Committee (IACUC) of the University of Puerto Rico (protocol no. A6850108) and by the University of Exeter School of Psychology’s Ethics Committee. We studied 100 free-ranging rhesus macaques living on the Cayo Santiago island (mean age = 10.6 years, 66 females and 34 males). This population is managed by the Caribbean Primate Research Center (CPRC) and was initially founded by the introduction of 409 rhesus macaques brought from India in the 1930s. Given that the current population is nearly 1700 individuals, which is well above the sustainable capacity based on demographic models, the CPRC has implemented a population management programme, which has led to the removal of animals via euthanasia. In this study, we used faecal samples collected during necropsy from 70 individuals that were removed and from another 30 individuals that were not part of the removal programme.

### Subjects and Study Site

We studied free-ranging rhesus macaques living on the Cayo Santiago island, Puerto Rico. Animals in this population are provisioned daily with commercial monkey chow and browse on natural vegetation, while water is supplied ad libitum from rainwater collection troughs. The island is predator-free and there is minimal medical intervention. In our study, we opportunistically collected parasite data from individuals that were scheduled for removal by the colony management. Given that the mean annual population growth rates of the Cayo Santiago macaques are higher than those of wild rhesus populations, live capture and removal of individuals have been implemented in this population since 1956. In 2016 and 2018, one entire social group of animals was removed in each of those 2 years. In the year leading up to their removal, we collected behavioural data on subadult and adult macaques (i.e. 4 years old or more) from those groups. Animals were removed between October and November in the respective year. Our final data set comprised 70 individuals from groups scheduled for removal (groups HH and KK) plus another 30 individuals from a group that is still on Cayo Santiago (group V). In total, we studied 100 animals (66 females and 34 males) between the ages of 4 and 26 years (mean = 10.6 years). Each of the social groups represents a single year of data. Macaques from groups KK and V were sampled after they experienced Hurricane Maria, which made landfall in September 2017, while group HH was removed a year before this event (details about groups sampled, sample size and data collection can be found in Table 1).

### Behavioural Data Collection

Behavioural data were collected using three protocols (Altmann, 1974): 5 min focal animal sample for group HH, group-wide scan sampling for group KK and event sampling for group V. Different protocols were required due to major ecological and global events (Hurricane Maria and the Covid-19 pandemic) that impacted the field station during the study. All data were collected by two experienced observers on all individuals in each group. Group HH was sampled using a previously established protocol for focal sampling (Brent et al., 2013) that allowed us to record detailed information on social interactions. Specifically, we recorded state behaviours (i.e. resting, feeding, travelling) along with grooming and agonistic interactions. Spatial proximity (i.e. sitting within 2 m of the focal animal) was recorded through scans at the beginning and end of each focal follow. For each of these records, the identities of the focal animal and social partner(s) and the specific behaviour were registered. Agonistic interactions included threat and submissive behaviours (i.e. avoid, lean, fear grimace) along with contact and noncontact aggression. Group KK was sampled using scan sampling due to constraints following the animals on the island and limited access to electricity for data collection devices because of the damage caused by Hurricane Maria. For this group, we recorded state behaviours, affiliative (i.e. grooming and proximity) and agonistic interactions between all visible individuals at 15 min intervals. Group V was sampled using event sampling because of restrictions on access to the field site during the COVID-19 pandemic as researchers were allowed in the field for half-days and only every 2–3 days. Specifically, we only recorded information on agonistic encounters, focusing on all the aggressive interactions described above. Group V thus only appears in our analyses of social status, not of relationship type or quality.

### Parasite Data Collection and Identification

We collected faecal samples opportunistically in the field from animals living in group V and in the laboratory during necropsy for animals belonging to groups KK and HH (details in Table 1). Samples from V were collected a few minutes after defecation within the period of behavioural data acquisition. Samples from HH and KK were collected from the intestines of individuals during necropsy during the same month or 1 month after behavioural data collection was finished. We collected 4–5 g of faecal material per animal in 50 ml falcon tubes, which were then filled with 20 ml of 10% formalin buffer. Each faecal sample was homogenized and stored at room temperature in the facilities of the CPRC. In total, we collected 100 samples from 100 individuals: 54 samples were collected before the hurricane (34 females, 20 males) and 46 samples after the hurricane (32 females, 14 males).

**Figure 1.**
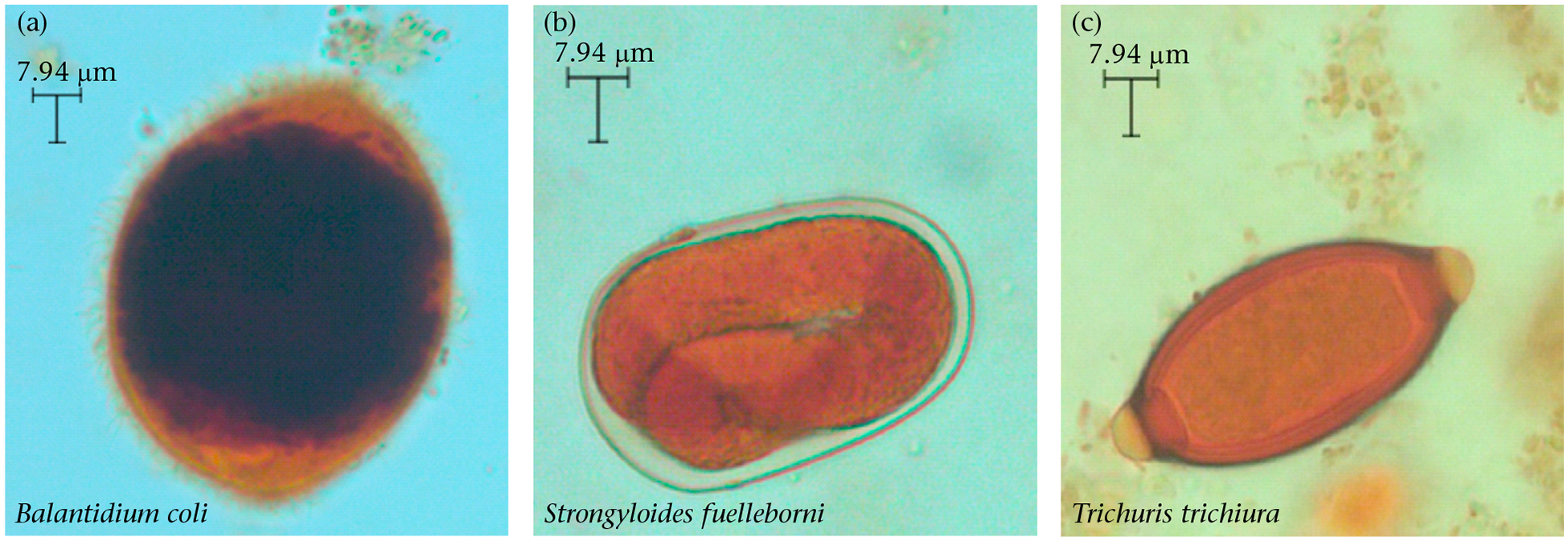
Most prevalent parasite species found in the faecal samples of the Cayo Santiago macaques, including (a) a protozoan and (b, c) two nematodes. Photos were taken with a light microscope camera.

We used the formalin ethyl-acetate sedimentation technique to extract faecal parasites (Centers for Disease Control and Prevention, 2016; Gillespie, 2006). Briefly, we filtered the formalized faeces and centrifuged 5 ml of sample diluted in 10 ml of formalin at 1830 rpm for 10 min. The supernatant was then discarded leaving 0.5–1 ml of sediment. We diluted the sediment again in 10 ml of formalin and added 4 ml of ethyl acetate. Samples were then homogenized and centrifuged again at 1830 rpm for 10 min. Finally, the supernatant was discarded, and the sediment was preserved for analysis by adding 10 ml of formalin buffer. We identified parasite taxa by direct observation based on their morphology (e.g. shape, colour, size; Despommier et al., 2017; Garcia et al., 2018). Larvae were rarely seen, and we, therefore, identified the presence of helminths based on the morphology of eggs. We estimated the number of parasite eggs or cysts using a wet mount procedure, for which an aliquot (ca.100 μl) of the homogenized suspension was placed on a microscopic slide, stained with 5% iodine solution and examined at 10x and 40x magnification. We used the average of two replicated counts per animal to estimate the number of parasite eggs or cysts that were infecting a host per sample (MacIntosh et al., 2012). We identified five parasite taxa across all samples including one protozoan (*Balantidium coli*) and four nematodes (*Ascaris lumbricoides, Ancyclostoma* spp., *Strongyloides fuelleborni*, *Trichuris trichiura*). For our analyses, we focused on the three most prevalent parasites (Fig. 1; *B. coli*, *T. trichiura* and *S. fuelleborni*; details on prevalence are presented in the Results), which were also detected in previous studies in the Cayo Santiago population (File & Kessler, 1989; Knezevich, 1998). We estimated two measures of infection per host from the samples: the presence of infection per parasite taxa (yes/no) and the intensity of infection (i.e. count of eggs or cysts of a given parasite in infected hosts; Rozsa et al., 2000). Despite being among the most common parasites found in the samples, *T. trichiura* and *S. fuelleborni* were not prevalent enough to allow us to test for effects on the intensity of infection (*N* infected = 23 and 24, respectively). Thus, we only examined the presence/absence of infection for these parasites.

### Social Status

To determine an individual’s social status, we computed dominance hierarchies by group and separately for males and females (Brent et al., 2017; Chancellor & Isbell, 2008; Kulik et al., 2015). Our approach is based on the fact that, in this species, males and females acquire social status differently. Females are philopatric and form maternally inherited stable linear dominance hierarchies, where daughters usually acquire rank just below their mothers (Chikazawa et al., 1979). In contrast, males typically disperse from the natal group and acquire rank in the new group by physical contest and tenure (Manson, 1995). We built independent hierarchies for the three social groups using dominance matrices filled with the outcomes of win - loss agonistic encounters from focal/scan sampling and ad libitum observations (De Vries, 1998). Because females typically acquire rank below that of their mothers, we used information on maternal relatedness to complement behavioural observations to resolve the gaps in the female hierarchy. That is, if the position of a female in the hierarchy could not be fully defined based on the agonism data alone, we used maternal rank inheritance and the rule of youngest ascendancy to resolve her position (Datta, 1988). Given that we collected only ad libitum data for group V, we confirmed our rank assignments by comparing them with rank assignments for this group from the previous year (2019), which were based on ad libitum and focal data. To account for variation in group sizes, dominance rank was defined as the percentage of group mates from a subject’s sex that they outranked, where 100% corresponded to the highest-ranking animal (Madlon-Kay et al., 2017).

### Weak and Strong Social Relationships

**Figure A1.**
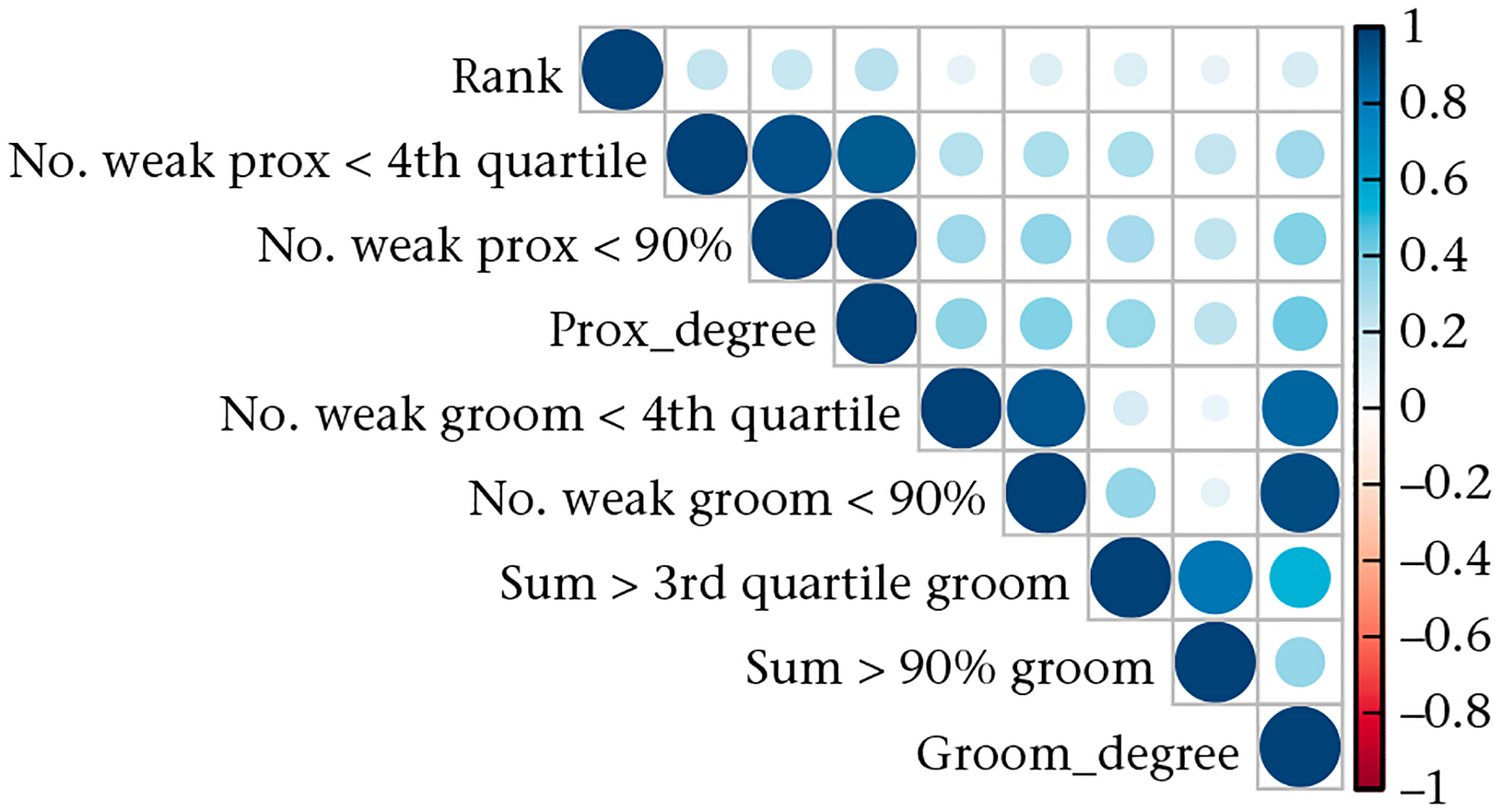
Correlation matrix between network metrics computed with our data set obtained from 50 draws from the posterior distributions. Colour reflects the Pearson correlation coefficient. Rank = social status, no. weak prox < 4th quartile = number of weak proximity partners considering as threshold the upper quartile (75%), no. weak prox < 90% = number of weak proximity partners considering as threshold the 90% quantile, prox_degree = number of proximity partners, no. weak groom < 4th quartile = number of weak grooming partners considering as threshold the upper quartile (75%), no. weak groom < 90% = number of weak grooming partners considering as threshold the 90% quantile, sum > 3rd quartile groom = strength to strong partners considering as threshold the upper quartile (75%), sum > 90% groom = strength to strong partners considering as threshold the 90% quantile, groom_degree = number of grooming partners.

Using proximity and grooming interactions, we constructed social networks for groups where we had data on affiliative interactions (*N* = 2 groups). We included all nonjuveniles for which we had data: all adult animals from group KK and adults plus sub-adults for group HH. We built separate networks for each interaction type. We focused on two network metrics that allowed us to delineate the extent to which individuals participated in different relationship types: an individual’s number of weak connections and their frequency of interaction (i.e. their relationship strength) with strong partners. However, we note these measures are correlated with related network metrics (Fig. A1) that have been explored in other studies looking at the relationship between sociality and health (e.g. degree: Duboscq et al., 2016; Pavez-Fox et al., 2021). Weak connections were quantified as the number of social partners with whom an individual engaged in infrequent affiliative interactions, while the frequency of affiliative interactions with strong partners quantified the time invested in strong relationships (Ellis et al., 2019; Schulke et al., 2022). The thresholds used to establish weak and strong partnerships are explained below. We quantified the number of weak partners for the two interaction types: grooming and proximity. For the strength of strong partners, we decided to focus only on grooming interactions, as they reflect an active partner choice (Henzi & Barrett, 1999), which might not be the case for proximity interactions.

We constructed one grooming and one proximity network for each group year using the bisonR package (Hart et al., 2022, 2023). This package allowed us to account for uncertainty in the edges connecting individuals in the network based on how often they were sampled and, importantly, propagate this uncertainty to subsequent analyses. In all our networks, nodes represented individuals and edges represented the undirected rate of grooming or proximity between a dyad. More specifically, for the proximity networks, an edge represented the number of times a pair of individuals were observed in proximity relative to the total observation effort for the dyad (i.e. total scans individual A + total scans individual B). For the KK grooming network, an edge between individuals represented the number of scan records a dyad engaged in grooming interactions relative to their total observation effort (i.e. total scan records individual A + total scan records individual B). Because HH was sampled using focal follow, grooming data were in the form of duration. To make both grooming networks more similar, we converted duration data to count data in the HH grooming network. For this, grooming observations during a given focal record were considered binary (i.e. whether the focal individual engaged in grooming or not). By doing so, the edges in the HH grooming network resemble the edges in the KK network where the numerator corresponded to the number of times a dyad was observed grooming and the denominator, to the observation effort for the dyad (i.e. total number of focal records of individual A + total number of focal records of individual B). In all our networks, we estimated the uncertainty around edge weights fitting a Bayesian ‘edge model’ where the prior was a conjugate beta distribution (bounded between 0 and 1), which was updated in every run with the observational data (see Hart et al., 2022, 2023 for more information). From these Bayesian networks, we obtained a posterior distribution of edge weights for each dyad, from which we extracted 100 draws to use in subsequent analyses.

**Figure A2.**
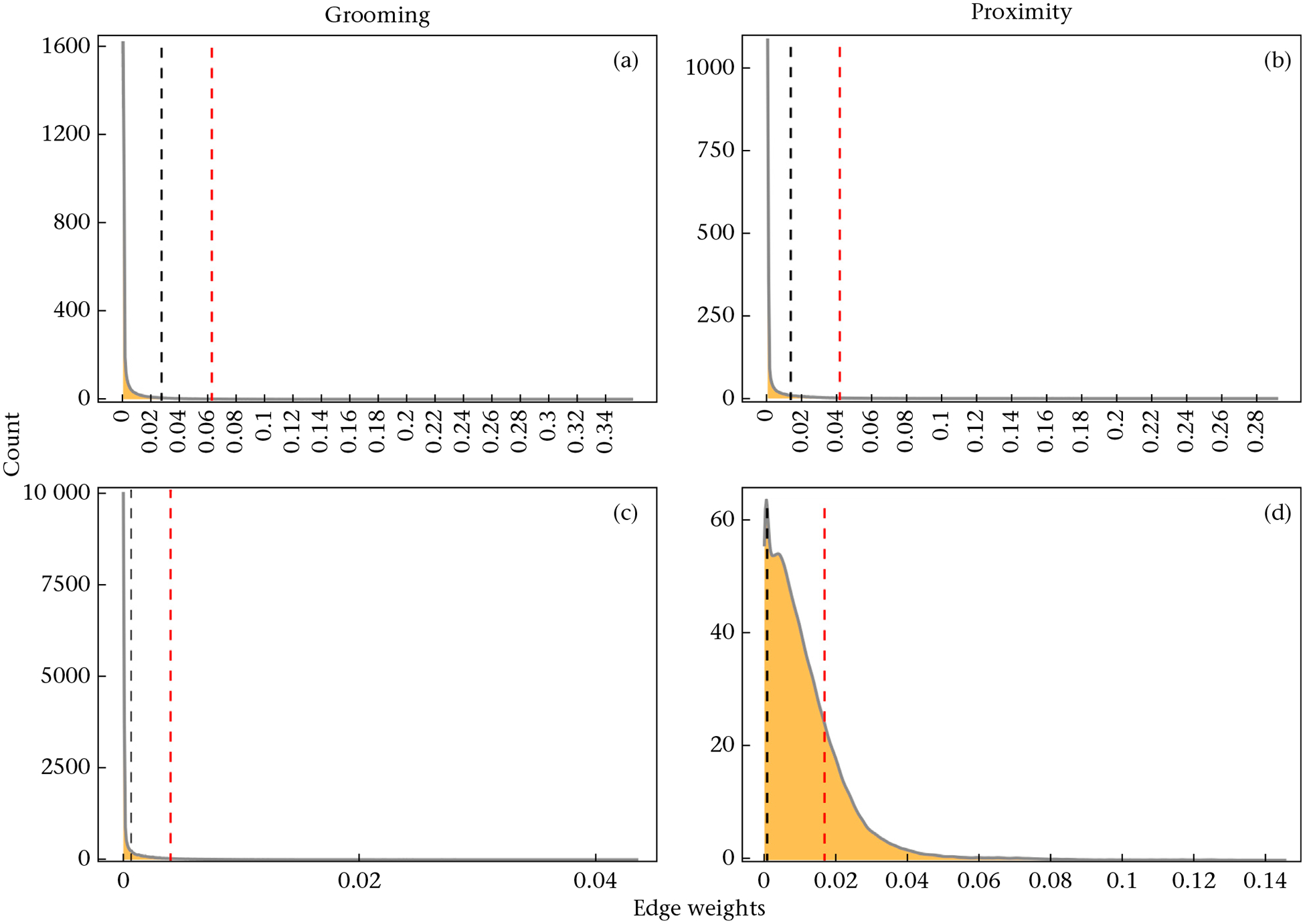
Thresholds used to define existent connections (black dashed line) and strong partners (red dashed line) for (a, c) grooming and (b, d) proximity networks in groups (a, b) HH and (c, d) KK generated with BISoN.

Networks generated with the bisonR package include edges between all dyads by default, as it assumes nonzero probability for all potential interactions, even if that probability is exceedingly small (Hart et al., 2022). To compute the number of weak partners, we defined a threshold that allowed us to differentiate dyads that did interact versus those that were not observed interacting based on the minimum empirical edge weight in each network. That is, for each of the posterior samples, dyads with an edge weight above or equal to the minimum empirical edge weight were kept and those below that value were excluded from the computation of network metrics. Strong partners were defined as dyads that had an edge weight within the upper quantile (i.e. 75% and above) of all the existent connections in a given network, while weak partners were those dyads that had edge weight values below this quantile (see Fig. A2 for visualization of both thresholds used on each network). An individual’s number of weak partners was the number of edges they had that were classed as ‘weak’. An individual’s strength to strong partners was computed by summing the weights of their edges that were classed as ‘strong’ connections. All network metrics were set to range between 0 and 10 by dividing an individual’s metric by the maximum value of that metric for the group and multiplying it by 10 (Ellis et al., 2019). By doing so, we accounted for possible group differences attributed to sampling methods because the network metrics were scaled relative to other individuals within a group. Our Bayesian network approach and within-group standardization allowed us to combine data collected using different sampling protocols into a single analysis. Specifically, by modelling the uncertainty around each edge in a network, we can explicitly control for differences in sampling effort within a network. This is particularly useful for scan sampling, where some individuals might be more likely to be sampled than others. By standardizing within a group, we make sure an individual’s connections are relative to other members of the group that were sampled in the same way. This matters because sampling differences can drive apparent differences in connectivity. For example, if proximity interactions are more likely to be captured with scan sampling than focal follow, individuals may appear more connected in a proximity network using the former sampling protocol than the latter.

### Statistical Analyses

All statistical analyses were carried out in R v4.3 using the brms package for Bayesian statistics (Burkner, 2017). For the models on the presence/absence of infection, the dependent variables were the binary presence of a given parasite species in a host. For models on the intensity of infection, the dependent variable was the count of *B. coli* cysts in infected hosts (Rozsa et al., 2000; *N* infected = 60). We modelled presence/absence of infection using linear regressions with a binomial distribution (‘Bernoulli’ in brms environment) and *B. coli* infection intensity using a negative binomial distribution. Model specifications are described below and included in Table A1.

### Social status, parasite infection and the impact of the hurricane

To determine whether the presence of infection was influenced by an individual’s social status and/or by Hurricane Maria, we ran one model per parasite species (three models in total). As predictors, we included an individual’s social status, the hurricane status, where 0 = sampled before and 1 = sampled after the hurricane, along with the age and the sex of the animal. Because age and sex might influence the relationships between social status and the impact of the hurricane on infection risk (Albery et al., 2023; Ginaldi et al., 2001; Tidiere et al., 2020), we tested for interactive effects and retained those for which evidence of an effect (i.e. credible interval not overlapping zero) was detected. We also included a fixed effect for the season when the sample was collected (rainy versus dry season) to account for variations in precipitation and temperature that might influence parasite dynamics (Altizer et al., 2006). The rainy season was considered to be the period between April and November and the dry season the period between December and March (Quintero et al., 2010). Using the same predictors, we assessed the influence of the hurricane on parasite intensity (1 model for *B. coli*).

### Affiliative relationships and risk of infection

Given that animals from group V did not have behavioural observations on affiliative interactions, the analyses that included social network metrics had a smaller sample size (*N* = 70). This reduction in sample size resulted in a smaller number of individuals infected with *B. coli* (*N* = 36) and only a few animals with parasite data after the hurricane (*N* = 16), precluding our ability to test the possible effect of affiliative relationships on infection intensity and how that might be modified by Hurricane Maria. All the faecal samples for the remaining groups (HH and KK) were collected during the same period (between October and November in their respective years); therefore, season was not included in the models.

To test whether weak and strong relationships influenced the presence of infection we ran one model per parasite species per network metric (nine models in total). Given our restricted sample size, we decided to include a single network metric per model to be able to account for relevant confounders, such as age, sex and social status, without risks of overparameterization. Because of this same reason, we did not examine interactive effects among predictors.

### Model specifications

In all our models we used weakly informative priors, which are recommended over flat priors to avoid overfitting issues when sample sizes are small and no prior knowledge of the relationship between the dependent variable and predictors are assumed (McElreath, 2020). Specifically, we used a Student’s *t* distribution with a mean of 0, 5 degrees of freedom and a standard deviation of 2.5 for all our fixed effects. We opted for a Student’s *t* distribution as it is less sensitive to outliers or skewed data compared to a normal distribution. Using weakly informative priors that assign more weight to the absence of an effect (mean = 0) also helps to mitigate the need to account for multiple testing when repeated tests of the same data set are performed (Lemoine, 2019), as in our case. We *z*-scored all the continuous predictors to improve sampling efficiency and to match prior specifications for the intercept (mean-centred at 0). We assessed model convergence by examining the R-hat values (ca. 1), effective sample sizes (> 1000) and visual inspection of the chains. We checked the goodness of fit of the models by using the pp check function from the brms package, which allowed us to do posterior predictive checks by comparing the data from the posterior distribution of the models with the observed data. If interactive effects found, we used the emmeans R package (Lenth, 2022) to perform post hoc tests of significance. We report means as point estimates, standard error (SE) and 89% credible intervals of the posterior distribution. Evidence for an effect was determined based on the degree of overlap between the credible interval and zero (i.e. 89% nonoverlapping reflecting strong evidence for an effect). For post hoc tests, we report the median and the 89% highest posterior density interval (HPD).

## RESULTS

The most common parasite detected in our samples was a protozoan (*B*. *coli*), which was present in 60 of the animals sampled (60% prevalence) and two nematode species: *T*. *trichiura* (24% prevalence) and *S*. *fuelleborni* (23% prevalence). We also identified two other helminth taxa (*A*. *lumbricoides, Ancyclostoma* spp.), but these were rarely seen (detected once in the full data set) and thus not included in downstream analyses. Data on infection status per parasite species with age, sex and rank are depicted in Figs. A4–A6.

### Social Status, Parasite Infection and the Impact of the Hurricane

We found *B. coli* in 38 individuals before the hurricane (70% prevalence) compared to 22 animals after the hurricane (48% prevalence). *Strongyloides fuelleborni* was present in eight animals before the hurricane (15% prevalence) and 15 individuals after (33% prevalence), while *T. trichiura* had a prevalence of 15% before the hurricane (present in eight animals) and 35% after the hurricane (present in 16 individuals). No parasite eggs were found in the faeces of 25 of the 100 individuals at the time of data collection.

**Figure 2.**
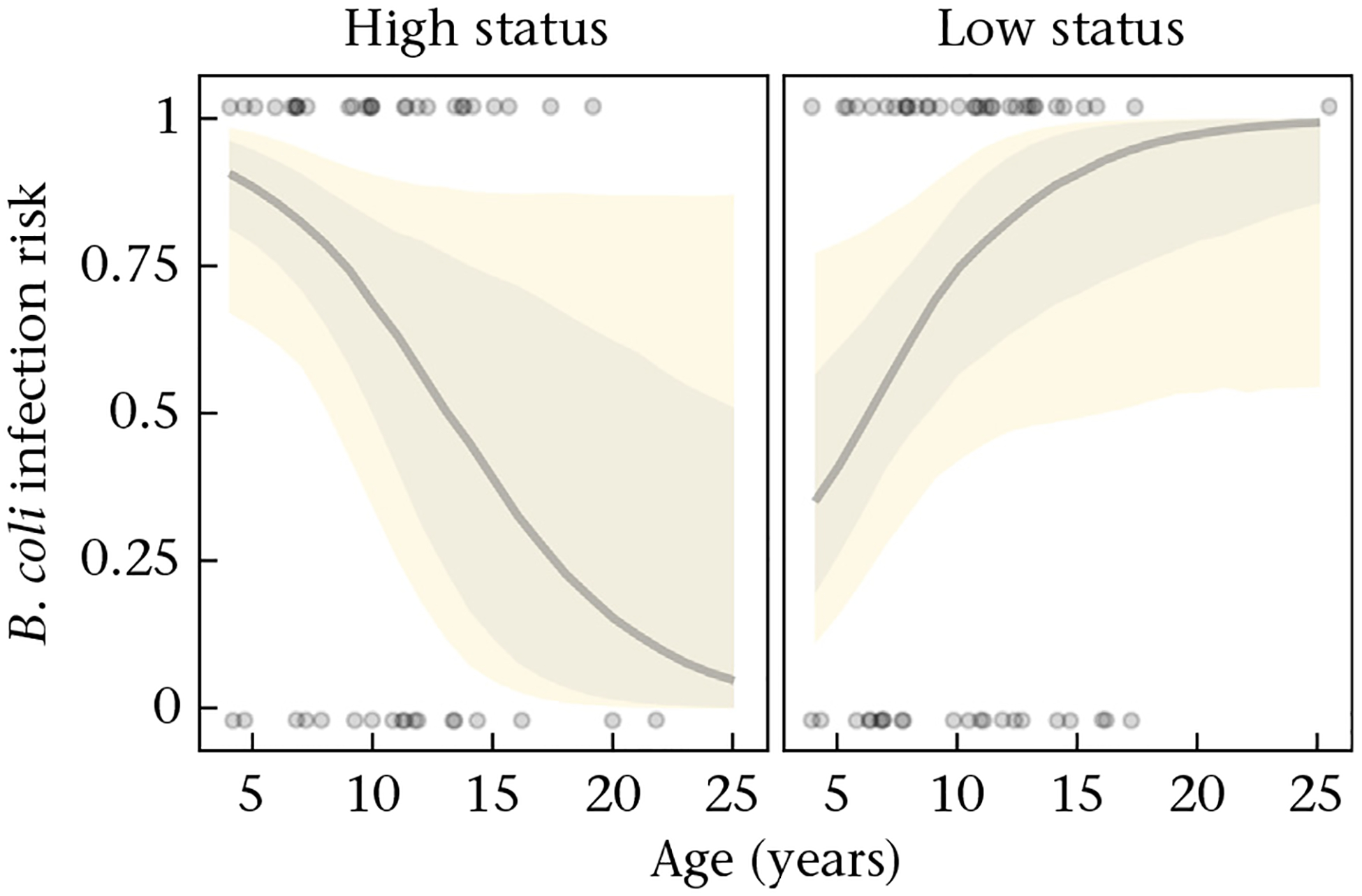
Risk of *Balantidium coli* presence of infection as a function of age and social status. For visualization, social status was categorized by selecting the 20th and the 80th percentiles depicting low and high status, respectively. Solid grey lines represent the median and the shaded areas illustrate the 50% (darker) and 80% (lighter) credible intervals computed from 100 draws from the posterior distribution. Data are depicted with grey points (1: infected; 0: noninfected).

We found effects of social status on *B. coli* infection risk that were dependent on animal age. Older high-status individuals were less likely to be infected with this protozoan than younger high-status (Fig. 2; post hoc test high-status: log odds age = −0.215, 89% HPD = −0.42, −0.02; Table 2) and older low-status animals (post hoc test: log odds low versus high status = 0.38, 89% HPD = 0.084, 0.68). Conversely, older low-status animals had a higher risk of *B. coli* infection than younger low-status macaques (Fig. 2; post hoc test low status: log odds age = 0.173, 89% HPD = 0.03, 0.34; Table 2). These estimates for age were obtained by categorizing social status into high status for individuals that outranked 80% and low status for those that outranked 20% of the same-sex members of their group.

**Figure 3.**
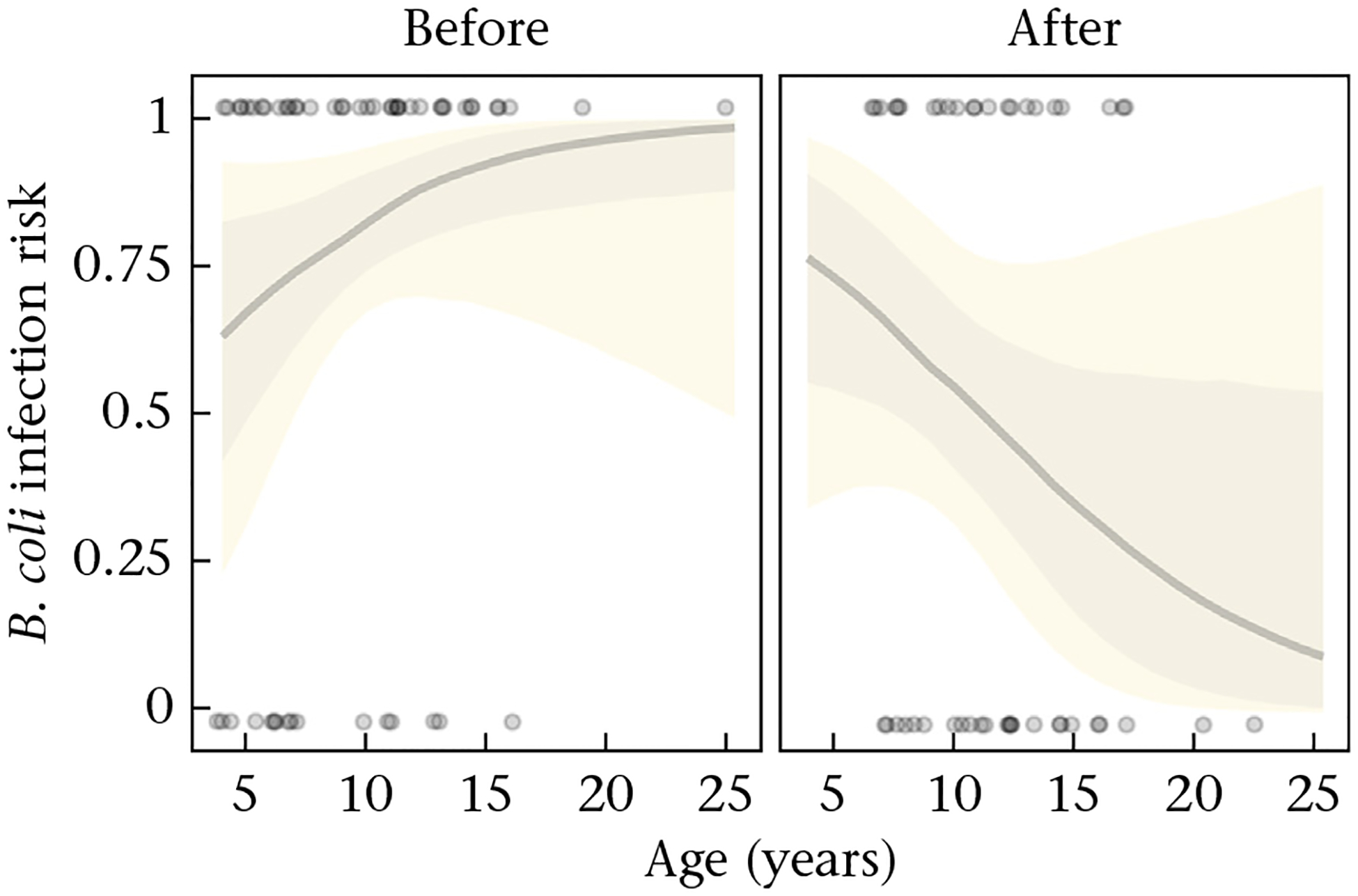
Risk of *Balantidium coli* presence of infection as a function of age and hurricane status (before and after the hurricane). Solid grey lines represent the median and the shaded areas illustrate the 50% (darker) and 80% (lighter) credible intervals computed from 100 draws from the posterior distribution. Data are depicted with grey points (1: infected; 0: noninfected).

The hurricane influenced infection risk in a parasite-specific fashion; however, these results should be taken with caution given the lack of longitudinal data. Macaques were less likely to be infected with *B. coli* and had a lower intensity of infection with this protozoan after the hurricane compared to before the hurricane (Table 2). The presence of *B. coli* infection was also predicted by the hurricane independent of the sex and the season but dependent on an individual’s age. That is, *B. coli* infection risk was higher in older than younger animals before the hurricane (Fig. 3; post hoc test: log odds prehurricane = 0.18, 89% HPD = 0.04, 0.33). After the hurricane, there was a trend for *B. coli* infection risk being lower in older than younger animals, but the evidence was weak (Fig. 3; post hoc test: log odds posthurricane = −0.14, 89% HPD = −0.31, 0.01; Table 2). Nematode infection was also influenced by the hurricane; macaques were more likely to be infected with *S. fuelleborni* and *T. trichiura* after the hurricane than before (Table 2).

We also found differences in infection risk with sex independent of social status and the hurricane. Males were at higher risk of *T. trichiura* infection than females (Table 2). Altogether our results show that (1) older and low-status individuals had the highest risk of protozoan infection, (2) before the hurricane there was a greater risk of protozoan infection but a lower risk of nematode infection compared to after the hurricane, and (3) males were more at risk of nematode infection than females.

### Affiliative Relationships and Risk of Infection

**Figure A3.**
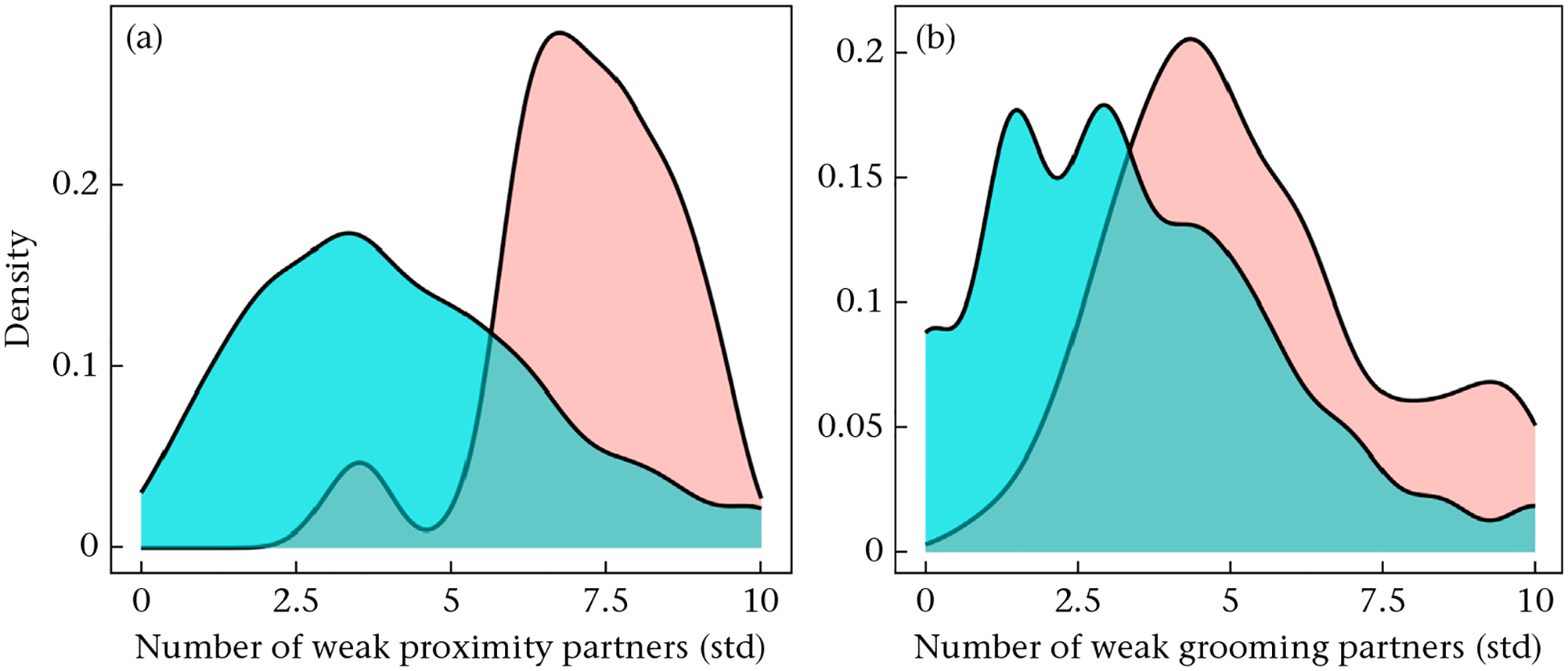
Distribution of the number of (a) weak proximity partners and (b) weak grooming partners obtained from 20 draws from BISoN networks for the group sampled before (blue) and after (red) the hurricane. Std: standardized within groups by dividing each value by the maximum and multiplying it by 10.

**Figure A4.**
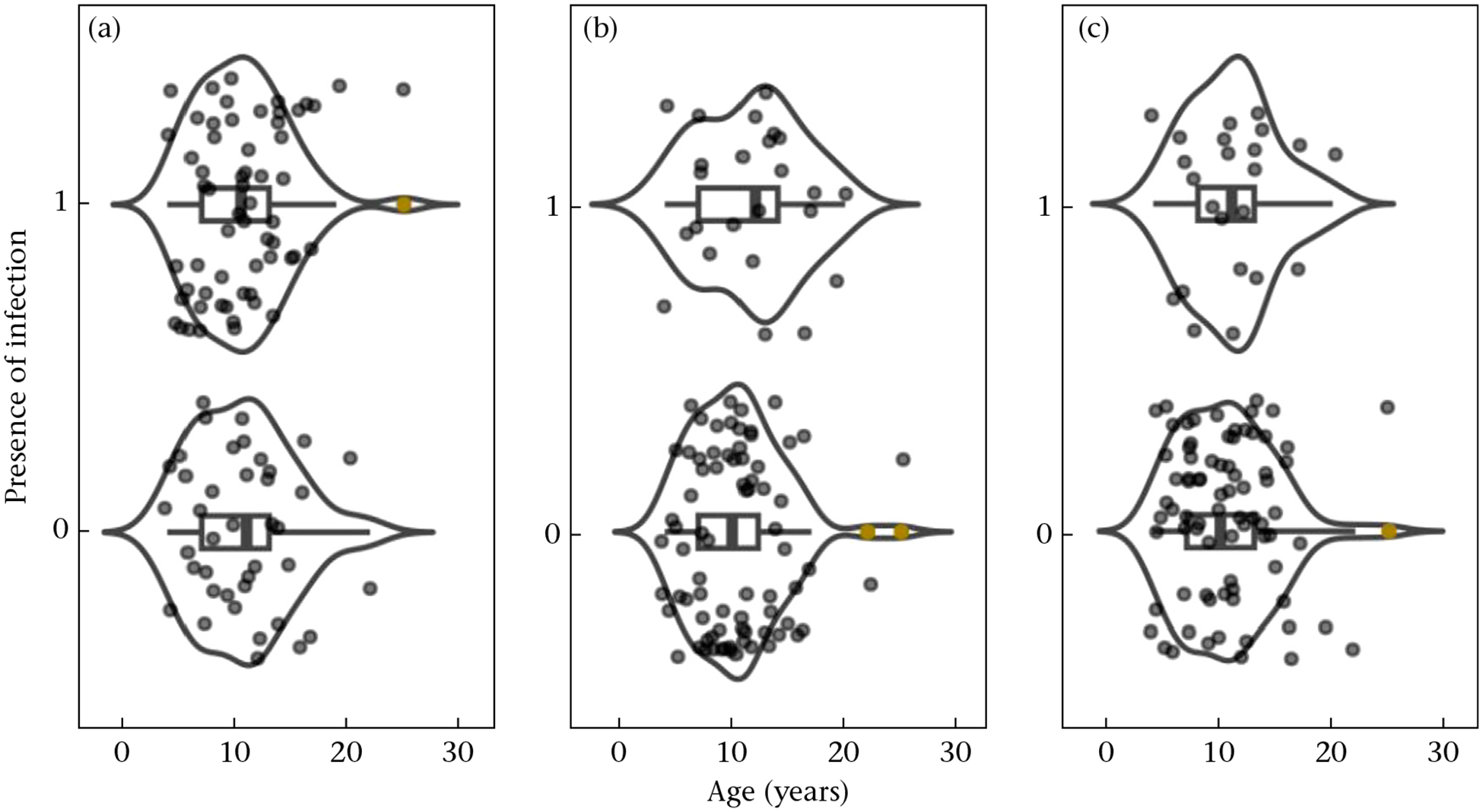
Data depicting the distribution of infected (1) and noninfected (0) individuals by age for (a) *B. coli*, (b) *T. trichiura* and (c) *S. fuelleborni*. Each point represents an individual with outliers in brown. The solid line surrounding the points depicts the probability density across age values. The box plots show the median and 25th and 75th percentiles; the whiskers indicate the values within 1.5 times the interquartile range.

**Figure A5.**
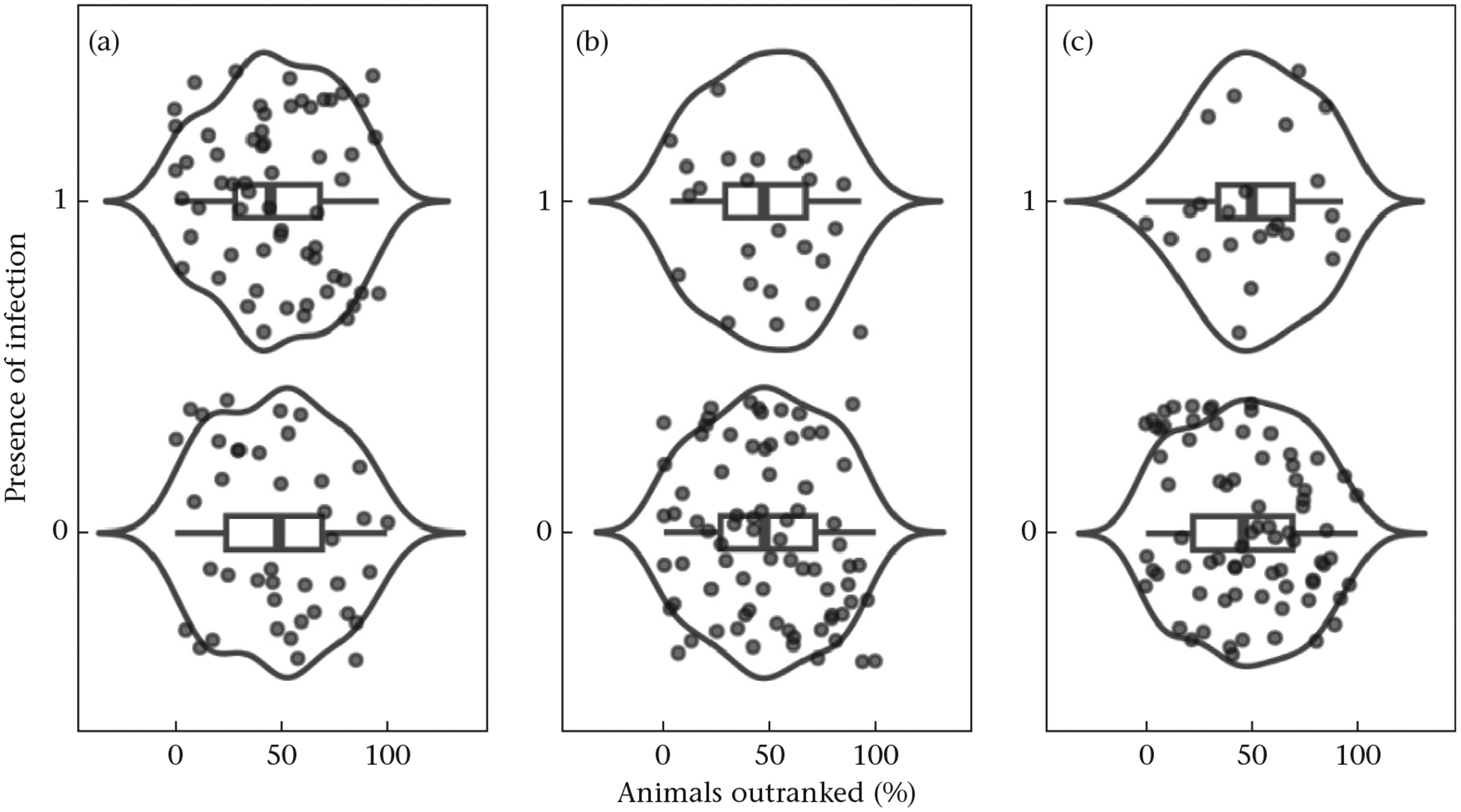
Data depicting the distribution of infected (1) and noninfected (0) individuals by rank for (a) *B. coli*, (b) *T. trichiura* and (c) *S. fuelleborni*. Each point represents an individual. The solid line surrounding the points depicts the probability density across rank values. The box plots show the median and 25th and 75th percentiles; the whiskers indicate the values within 1.5 times the interquartile range.

**Figure A6.**
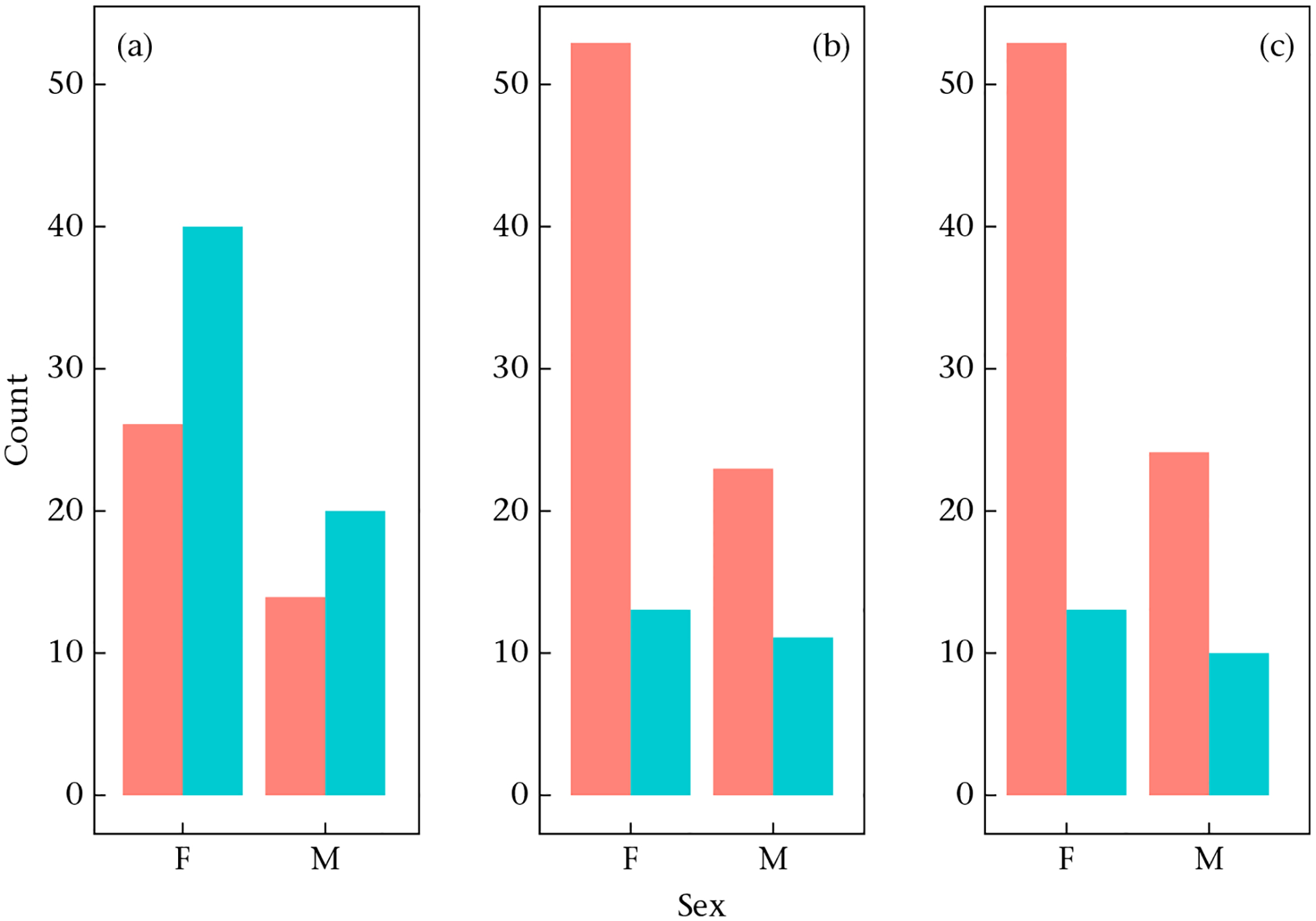
Data depicting the distribution of infected individuals by sex (F: female; M: male) for (a) *B. coli*, (b) *T. trichiura* and (c) *S. fuelleborni*. Infected individuals are depicted in blue and noninfected animals in red.

Among all the measures of affiliative relationships and parasites tested, we only found evidence for a negative relationship between the number of weak proximity partners and, to a lesser extent, weak grooming partners on the presence of *B. coli* infection (Table 3). However, these results should be taken with caution. Given our limited sample size for these models, we could not account for the effect of the hurricane. The results in Table 2 show that the presence and intensity of *B. coli* infection decreased after the hurricane. Additionally, individuals sampled after the hurricane seemed to have more weak proximity partners compared to individuals sampled before the hurricane in our data set (Fig. A3). These results together suggest that the reduced infection risk with *B. coli* in individuals with more weak proximity partners is likely confounded by the effect of the hurricane on the prevalence of this parasite.

## DISCUSSION

In this study, we explored the relationship between sociality and faecal parasite infection in free-ranging female and male rhesus macaques. Using cross-sectional data, we found evidence for an effect of social status on infection risk that was dependent on an individual’s age. Older high-status animals were less likely to be infected than younger high-status animals, while the opposite occurred in low-status macaques. In addition, we found that environmental changes associated with Hurricane Maria influenced the risk of infection with protozoa and nematode parasites independent of an individual’s social status but in an age-specific manner. When exploring the role of social interaction type and quality on infection risk, we found that macaques with more weak proximity partners had a reduced risk of protozoan infection; however, this relationship was likely driven by independent effects of Hurricane Maria on the social dynamics and the prevalence of parasites in this population. Our results show that social capital in the form of social status can promote health by mitigating the costs of faecal parasite infection.

Consistent with literature suggesting that high-status animals are often more exposed to parasites (Habig & Archie, 2015; MacIntosh et al., 2012), we found that younger high-status animals had a higher risk of *B. coli* infection than low-status animals of similar ages. In several vertebrate species, high-status individuals had a higher parasitic load from contact and environmentally transmitted parasites, which is posited to be due to greater exposure given their priority of access to resources (Habig et al., 2018) and immunosuppression due to the high energetic demand associated with reproductive effort (Smyth et al., 2016). Given that this effect was only evident at younger ages, it is likely that dominant rhesus macaques were more exposed to parasites due to taking most of the food, which might increase their risk of infection with parasites transmitted via the faecal - oral route (Halvorsen,1986). In older macaques, we found the opposite relationship, where low-status animals were at higher risk of protozoan infection than high-status macaques. This could reflect increased susceptibility due to immunosenescence (Ginaldi et al., 2001) or be the consequence of higher exposure in low-status animals. For instance, *B. coli* is commonly found in contaminated water (Schuster & Ramirez-Avila, 2008), such as standing water puddles. Macaques in this population usually soak monkey chow in such puddles before chewing them (personal observation), possibly to make them softer. This behaviour might be more common among older animals if they have weakened bite strength. However, high-status animals may be more likely to access clean water provisioned in drinking troughs (Balasubramaniam et al., 2014), unlike low-status animals which might rely more on contaminated puddles making them more exposed to *B. coli* infection. Differences in susceptibility could also explain higher infection risk in old low-status individuals; no evidence has been found in this population, however, for an effect of social status on immunosenescence (Sanchez Rosado et al., 2024) or in immune function (Pavez-Fox et al., 2021). These results suggest that differences in infection risk with social status and age are most likely driven by changes in exposure to parasites.

Our results suggest that environmental changes associated with a major disaster are not always associated with a greater risk of parasite infection, as it may also depend on the life cycle of the parasite under study. Hurricane Maria had an important effect on parasite infection in the Cayo Santiago population. The presence and the intensity of infection with the protozoan *B. coli* were higher before the hurricane than after, while the opposite was true for infection risk with the nematodes *S. fuelleborni* and *T. trichiura*. While we cannot disregard these differences arising from group membership (e.g. differences in home range, percentage of natural vegetation in the diet), these results suggest that changes in the environment as a consequence of the hurricane have made the island less or more suitable for these parasites. For *B. coli*, the optimal environmental conditions for the infective stage (i.e. cysts) are humid areas protected from direct sunlight (Schuster & Ramirez-Avila, 2008) which were very scarce after the hurricane given the massive loss of vegetation cover (Testard et al., 2021; Watowich et al., 2022). Conversely, nematode eggs may be more resistant to the dry conditions on the island after the hurricane than protozoan cysts (Escabi Ruiz, 2022; Mkandawire et al., 2022; Salih, 1981). It is likely that macaques were more exposed to nematode parasites after the hurricane due to being more restricted in space because of the debris on the island and due to seeking out limited shady spots (Testard et al., 2021). This may have led to more overlap with contaminated (defecated) areas and to increased social contact between individuals, potentially facilitating the social transmission of parasites.

Although social status influenced protozoan infection risk in our study, we did not find evidence of a buffering effect of social status on the presence of this parasite in the face of the hurricane. One possible explanation is that given the low prevalence and intensity of infection with *B. coli* after the hurricane, our ability to detect changes in the presence of infection with social status by examining a single sample per animal is limited. For instance, inconsistencies in the shedding of cysts in faeces may have led us to not identify individuals as infected when they really were (false negatives). We can also not disregard differences attributed to group membership given the use of cross-sectional instead of longitudinal data to compare before and after the hurricane. Alternatively, because *B. coli* infection can have very detrimental health consequences in immunocompromised individuals (Schuster & Ramirez-Avila, 2008), it is possible that older low-status individuals infected before the hurricane did not survive the aftermath of the hurricane, leaving mostly animals that were resistant to this infection.

We found that macaques with more weak proximity and grooming partners were less likely to be infected with *B. coli*; however, this result might not hold if we account for differences in prevalence attributed to the hurricane. That is, rhesus macaques in our data set tended to have more weak proximity and grooming partners after the hurricane than before (Fig. A3), which is consistent with previous results in this population showing that increasing the number of affiliative partners might be a strategy to deal with the aftermath of the hurricane (Testard et al., 2021). Because the conditions after the hurricane might have been less suitable for *B. coli*, it is possible that the relationship between reduced infection risk and weak partners was confounded by the effects of the hurricane, which we were unable to account for statistically. Alternatively, we cannot disregard other explanations such as variation in exposure and susceptibility among individuals of different groups or a protective effect of sociality that is only apparent under extreme environmental conditions.

In line with previous studies suggesting that males suffer from higher parasitism than females (Moore & Wilson, 2002; Tidiere et al., 2020), we found that male rhesus macaques had a higher risk of *T. trichiura* infection. Why males are more likely to be parasitized than females can be explained by differences in susceptibility and exposure. Males are often more susceptible to infections than females due to differences in immunity regulation by sex steroids. For instance, oestrogens, which are in higher concentrations in females, have an immune-enhancing role, while males’ sex steroid testosterone has mainly an immunosuppressive effect (Tidiere et al., 2020). In addition, males from polygamous species, such as rhesus macaques, have been suggested to be more exposed to parasites due to higher rates of physical contact with potential mates (Habig et al., 2018). This could explain why we only found an effect on infection from the nematode *T. trichiura*, which is more likely to be transmitted by social contact than the protozoan *B. coli*.

Overall, our study provides some evidence for the role of social status, age and sex modulating infection risk, and highlights the relevance of considering environmental variation when studying patterns of infection in natural populations. It is important to mention that our study was opportunistic in nature and, as a consequence, suffers from limitations including sample size and the use of cross-sectional data. Future studies that incorporate longitudinal data on behaviour, faecal samples and even immunity might be better able to disentangle the relative contribution of the type and quality of affiliative relationships on infection risk in the macaques of Cayo Santiago and other systems. Notwithstanding, we believe the results found here highlight important avenues for future research looking at the interplay between sociality, infection risk and natural disasters.

## Figures and Tables

**Table 1:** Summary of the data used in this study

Group	Year	*N*	Parasite period	Behaviour	Period	Mean ± SD
HH	2016	54	Oct-Nov	Focal	Aug-Oct	1.46 ± 0.08
**Hurricane Maria 2017**						
KK	2018	16	Oct-Nov	Scan	Jan-Oct	538.1 ± 161.3
V	2020	30	Jan-Sep	Event	Jan-Dec	7.13 ± 4.6

Group: social group analysed; year: year for which that group was sampled; *N*: number of individuals analysed per group; parasite period: months when parasite data were collected (faecal samples (one per individual) from HH and KK were collected ‘post mortem’ from the intestines of the animals during necropsy, while samples from V were collected in the field during the behavioural data acquisition); behaviour: collection method for behavioural data where ‘focal’ indicates 5 min focal observation, ‘scan’ indicates 15 min group scans and ‘event’ indicates event sampling where only agonistic interactions were recorded; period: months when behavioural data were collected; mean ± SD: the average and standard deviation of behavioural sampling effort for each group (h), behavioural events and agonistic events, respectively.

**Table 2:** Model outputs predicting parasite infection in relation to social status and the hurricane

Predictor	Mean	SE	89% CI	Direction of effect
***B. coli* presence**				
Age	0.1	0.23	−0.26, 0.48	–
Rank	−0.05	0.22	−0.41, 0.31	–
Hurricane	−1.27	0.49	−2.07, −0.49	↓
Sex	−0.09	0.46	−0.82, 0.65	–
Season	−0.62	0.63	−1.66, 0.36	–
Age*Rank	−0.63	0.3	−1.14, −0.16	see Fig. 2
Age*Hurricane	−1.36	0.57	−2.32, −0.49	see Fig. 3
***B. coli* intensity**				
Age	0.18	0.16	−0.07, 0.45	–
Rank	0.08	0.18	−0.22, 0.37	–
Hurricane	−1.04	0.39	−1.65, −0.36	↓
Sex	0.23	0.31	−0.26, 0.75	–
Season	0.18	0.5	−0.64, 1.00	–
***S. fuelleborni* presence**				
Age	−0.01	0.27	−0.45, 0.42	–
Rank	0.21	0.27	−0.21, 0.65	–
Hurricane	1.23	0.59	0.3, 2.19	↑
Sex	0.52	0.52	−0.31, 1.37	–
Season	0.26	0.66	−0.78, 1.34	–
***T. trichiura* presence**				
Age	0.19	0.26	−0.23, 0.61	–
Rank	−0.12	0.27	−0.55, 0.31	–
Hurricane	0.94	0.57	0.04, 1.86	↑
Sex	0.91	0.53	0.06, 1.77	↑
Season	−0.52	0.66	−1.57, 0.55	–

89% CI: credible interval, ‘-’: no effect detected. ‘Hurricane’ considers prehurricane as the intercept. ‘Age’ and ‘Rank’ are continuous predictors, where higher values indicate older and higher-status animals, respectively. ‘Sex’ considers females as the intercept. ‘Season’ considers the dry season as the intercept. The main effects for ‘Age’, ‘Rank’ and ‘Sex’ were obtained from a model without interactions. Estimates for the intercept are included in Tables A2–A6.

**Table 3:** Model outputs predicting parasite infection in relation to affiliative relationships

Predictor	Mean	SE	89% CI	Direction of effect
***B. coli* presence**				
No. weak prox partners	−0.71	0.37	−1.37, −0.15	↓
No. weak groom partners	−0.69	0.44	−1.46, 0.00	↓
Str strong partners	−0.49	0.44	−1.26, 0.18	–
***S. fuelleborni* presence**				
No. weak prox partners	0.35	0.39	−0.28, 0.99	–
No. weak groom partners	0.37	0.42	−0.33, 1.06	–
***T. trichiura* presence**				
No. weak prox partners	0.34	0.4	−0.29, 0.98	–
No. weak groom partners	−0.06	0.42	−0.75, 0.61	–
Str strong partners	−0.04	0.44	−0.79, 0.63	–

Each row represents the estimates for measures of affiliative relationships run in separate models. Estimates for control variables (age, sex, rank) can be found in Tables A6–A15. 89% CI: credible interval, ‘-’: no effect detected. ‘no. weak prox partners’ and ‘no. weak groom partners’ indicate the number of weak grooming and proximity partners, respectively. ‘str strong partner’ indicates the strength of strong grooming partners.

## Data Availability

R code used for models and plots are available at https://github.com/MPavFox/Faecal-Parasites.git.

## Appendix

**Table A1:** Model specifications

Question	Model
Did the hurricane and social status influence the prevalence and intensity of parasite infections?	prev_coli ~ age*hurricane + rank*age + sex + season prev_coli ~ age + hurricane + rank + sex + season (main effect) prev_strong ~ age + hurricane + rank + sex + season prev_trichu ~ age + hurricane + rank + sex + season int_proto ~ age + hurricane + rank + sex + season
Did the number of weak connections an individual had predict infection risk?	prev_coli ~ weak_groom + sex + age + social status prev_strong ~ weak_groom + sex + age + social status prev_trichu ~ weak_groom + age + sex + social status prev_coli ~ weak_prox + sex + age + social status prev_strong ~ weak_prox + sex + age + social status prev_trichu ~ weak_prox + age + sex + social status
Did the frequency of interaction with strong partners predict infection risk?	prev_coli ~ groom_str + sex + age + social status prev_strong ~ groom_str + sex + age + social status prev_trichu ~ groom_str + age + sex + social status

prev_coli = presence/absence of *Balantidium coli*, prev_strong = presence/absence of *Strongyloides fuelleborni*, prev_trichu = presence/absence of *Trichuris trichiura*, hurricane = sampled before or after the hurricane Maria, sex = an individual’s sex, age = animal’s age when sampled, season = sampled collected during the wet or rainy season, int_prot = intensity of protozoan infection (*B. coli*), social status = rank of an animal relative to all same-sex members of its group, weak_groom = number of weak grooming connections, weak_prox = number of weak proximity connections, groom_str = strength to strong grooming partners.

**Table A2:** Model output for *B. coli* presence of infection: main effect (full data set)

Predictors	Infection risk		
	Log odds	SE	CI (89%)
Intercept	1.59	0.69	0.49–2.74
scaleage	0.10	0.23	−0.26–0.48
post_hurricane: post_hurricane1	−1.27	0.49	−2.07–−0.49
scaleoutrank_perc	−0.05	0.22	−0.41–0.31
sex: M	−0.09	0.46	−0.82–0.65
season: rainy	−0.62	0.63	−1.66–0.36
Observations	100		

scaleoutrank_perc = z-standardized percentage of same-sex group members outranked; scaleage = z-standardized age; post_hurricane1 = after the hurricane; sex: M = males; season: rainy = wet season; CI = credible interval.

**Table A3:** Model output for *B. coli* presence of infection: interactions (full data set)

Predictors	Infection risk		
	Log odds	SE	CI (89%)
Intercept	1.99	0.76	0.81–3.25
scaleage	0.75	0.38	0.18–1.39
post_hurricane: post_hurricane1	−1.50	0.54	−2.40–−0.66
scaleoutrank_perc	−0.19	0.25	−0.60–0.21
sex: M	0.25	0.49	−0.51–1.06
season: rainy	−0.81	0.66	−1.89–0.23
scaleage:post_hurricane1	−1.36	0.57	−2.32–−0.49
scaleage:scaleoutrank_perc	−0.63	0.30	−1.14–−0.16
Observations	100		

scaleoutrank_perc = z-standardized percentage of same-sex group members outranked; scaleage = z-standardized age; post_hurricane1 = after the hurricane; sex: M = males; season: rainy = wet season; CI = credible interval.

**Table A4:** Model output for *B. coli* intensity of infection (full data set)

Predictors	Infection intensity		
	Log mean	SE	CI (89%)
Intercept	2.52	0.55	1.64–3.42
scaleage	0.18	0.16	−0.07–0.45
scaleoutrank_perc	0.08	0.18	−0.22–0.37
post_hurricane: post_hurricane1	−1.04	0.39	−1.65–−0.36
sex: M	0.23	0.31	−0.26–0.75
season: rainy	0.18	0.50	−0.64–1.00
Observations	60		

scaleoutrank_perc = z-standardized percentage of same-sex group members outranked; scaleage = z-standardized age; post_hurricane1 = after the hurricane; sex: M = males, season: rainy = wet season; CI = credible interval.

**Table A5:** Model output for *S. fuelleborni* presence of infection (full data set)

Predictors	Infection risk		
	Log odds	SE	CI (89%)
Intercept	−2.22	0.78	−3.48–−1.01
Scaleage	−0.02	0.27	−0.46–0.40
post_hurricane: post_hurricane1	1.17	0.56	0.29–2.09
scaleoutrank_perc	0.21	0.27	−0.22–0.65
sex: M	0.54	0.52	−0.29–1.37
season: rainy	0.15	0.67	−0.92–1.23
Observations	100		

scaleoutrank_perc = z-standardized percentage of same-sex group members outranked; scaleage = z-standardized age; post_hurricane1 = after the hurricane; sex: M = males, season: rainy = wet season; CI = credible interval.

**Table A6:** Model output for *T. trichiura* presence of infection (full data set)

Predictors	Infection risk		
	Log odds	SE	CI (89%)
Intercept	−1.67	0.78	−2.93–−0.46
Scaleage	0.19	0.26	−0.23–0.61
post_hurricane: post_hurricane1	0.94	0.57	0.04–1.86
scaleoutrank_perc	−0.12	0.27	−0.55–0.31
sex: M	0.91	0.53	0.06–1.77
season: rainy	−0.52	0.66	−1.57–0.55
Observations	100		

scaleoutrank_perc = z-standardized percentage of same-sex group members outranked; scaleage = z-standardized age; post_hurricane1 = after the hurricane; sex: M = males, season: rainy = wet season; CI = credible interval.

**Table A7:** Model output for *B. coli* presence of infection as a function of the number of weak proximity partners (SNA data set)

Predictors	Infection risk		
	Log odds	SE	CI (89%)
Intercept	0.56	0.34	0.03–1.13
scalestd_weak_prox	−0.71	0.37	−1.37–−0.15
Scaleage	0.48	0.31	0.01–1.02
scaleperc_rank	0.35	0.29	−0.09–0.83
sex: M	−0.66	0.60	−1.66–0.27
Observations	70		

scalestd_weak_prox = z-standardized number of weak proximity partners; scaleoutrank_perc = z-standardized percentage of same-sex group members outranked; scaleage = z-standardized age; sex: M = males; CI = credible interval.

**Table A8:** Model output for *B. coli* presence of infection as a function of the number of weak grooming partners (SNA data set)

Predictors	Infection risk		
	Log odds	SE	CI (89%)
Intercept	0.52	0.34	−0.01–1.10
scalestd_weak_groom	−0.69	0.44	−1.46–0.00
scaleage	0.37	0.30	−0.09–0.89
scaleperc_rank	0.27	0.29	−0.17–0.76
sex: M	−0.52	0.59	−1.50–0.40
Observations	70		

scalestd_weak_groom = z-standardized number of weak grooming partners; scaleoutrank_perc = z-standardized percentage of same-sex group members outranked; scaleage = z-standardized age; sex: M = males; CI = credible interval.

**Table A9:** Model output for *S. fuelleborni* presence of infection as a function of the number of weak proximity partners (SNA data set)

Predictors	Infection risk		
	Log odds	SE	CI (89%)
Intercept	−1.43	0.40	−2.11–−0.83
scalestd_weak_prox	0.35	0.39	−0.28–0.99
scaleage	−0.07	0.34	−0.65–0.45
scaleperc_rank	−0.05	0.32	−0.58–0.46
sex: M	−0.25	0.70	−1.40–0.86
Observations	70		

scalestd_weak_prox = z-standardized number of weak proximity partners; scaleoutrank_perc = z-standardized percentage of same-sex group members outranked; scaleage = z-standardized age; sex: M = males; CI = credible interval.

**Table A10:** Model output for *S. fuelleborni* presence of infection as a function of the number of weak grooming partners (SNA data set)

Predictors	Infection risk		
	Log odds	SE	CI (89%)
Intercept	−1.43	0.40	−2.12–−0.83
scalestd_weak_groom	0.37	0.42	−0.33–1.06
scaleage	−0.04	0.34	−0.60–0.48
scaleperc_rank	−0.03	0.32	−0.54–0.49
sex: M	−0.28	0.69	−1.43–0.81
Observations	70		

scalestd_weak_groom = z-standardized number of weak grooming partners; scaleoutrank_perc = z-standardized percentage of same-sex group members outranked; scaleage = z-standardized age; sex: M = males; CI = credible interval.

**Table A11:** Model output for *T. trichiura* presence of infection as a function of the number of weak proximity partners (SNA data set)

Predictors	Infection risk		
	Log odds	SE	CI (89%)
Intercept	−1.79	0.45	−2.56–−1.13
scalestd_weak_prox	0.34	0.40	−0.29–0.98
scaleage	0.16	0.33	−0.39–0.67
scaleperc_rank	−0.35	0.34	−0.91–0.19
sex: M	0.70	0.69	−0.40–1.84
Observations	70		

scalestd_weak_prox = z-standardized number of weak proximity partners; scaleoutrank_perc = z-standardized percentage of same-sex group members outranked; scaleage = z-standardized age; sex: M = males; CI = credible interval.

**Table A12:** Model output for *T. trichiura* presence of infection as a function of the number of weak grooming partners (SNA data set)

Predictors	Infection risk		
	Log odds	SE	CI (89%)
Intercept	−1.69	0.42	−2.42–−1.06
scalestd_weak_groom	−0.06	0.42	−0.75–0.61
scaleage	0.20	0.31	−0.31–0.71
scaleperc_rank	−0.25	0.33	−0.79–0.26
sex: M	0.48	0.66	−0.58–1.53
Observations	70		

scalestd_weak_groom = z-standardized number of weak grooming partners; scaleoutrank_perc = z-standardized percentage of same-sex group members outranked; scaleage = z-standardized age; sex: M = males; CI = credible interval.

**Table A13:** Model output for *B. coli* presence of infection as a function of the strength to strong grooming partners (SNA data set)

Predictors	Infection risk		
	Log odds	SE	CI (89%)
Intercept	0.46	0.33	−0.06–1.02
scalestd_topstr_groom	−0.49	0.44	−1.26–0.18
scaleage	0.32	0.29	−0.13–0.80
scaleperc_rank	0.27	0.28	−0.16–0.74
sex: M	−0.38	0.57	−1.32–0.51
Observations	70		

scalestd_topstr_groom = z-standardized strength to strong grooming partners; scaleoutrank_perc = z-standardized percentage of same-sex group members outranked; scaleage = z-standardized age; sex: M = males; CI = credible interval.

**Table A14:** Model output for *S. fuelleborni* presence of infection as a function of the strength to strong grooming partners (SNA data set).

Predictors	Infection risk		
	Log odds	SE	CI (89%)
Intercept	−1.35	0.38	−2.00–−0.78
scalestd_topstr_groom	0.04	0.42	−0.66–0.75
scaleage	−0.01	0.32	−0.54–0.49
scaleperc_rank	0.00	0.32	−0.51–0.51
sex: M	−0.42	0.67	−1.55–0.62
Observations	70		

scalestd_topstr_groom = z-standardized strength to strong grooming partners; scaleoutrank_perc = z-standardized percentage of same-sex group members outranked; scaleage = z-standardized age; sex: M = males; CI = credible interval.

**Table A15:** Model output for *T. trichiura* presence of infection as a function of the strength to strong grooming partners (SNA data set)

Predictors	Infection risk		
	Log odds	SE	CI (89%)
Intercept	−1.70	0.42	−2.42–−1.07
scalestd_topstr_groom	−0.04	0.44	−0.79–0.63
scaleage	0.20	0.32	−0.31–0.71
scaleperc_rank	−0.26	0.33	−0.80–0.26
sex: M	0.48	0.65	−0.57–1.53
Observations	70		

scalestd_topstr_groom = z-standardized strength to strong grooming partners; scaleoutrank_perc = z-standardized percentage of same-sex group members outranked; scaleage = z-standardized age; sex: M = males; CI = credible interval.
